# Plate osteosynthesis of single metacarpal fracture: WALANT technique is a cost-effective approach to reduce postoperative pain and discomfort in contrast to general anesthesia and wrist block

**DOI:** 10.1186/s12893-021-01362-5

**Published:** 2021-10-09

**Authors:** Yen-Chang Lin, Wei-Chieh Chen, Chun-Yu Chen, Shyh-Ming Kuo

**Affiliations:** 1 Department of Orthopaedics, Kaohsiung Veterans General Hospital, 386 Ta-Chung 1st Road, Kaohsiung City, Taiwan; 2 Department of Occupational Therapy, Shu-Zen Junior College of Medicine and Management, Kaohsiung City, Taiwan; 3 Department of Biomedical Engineering, I-Shou University, Kaohsiung City, Taiwan

**Keywords:** WALANT, Wide awake, No tourniquet, Metacarpal fracture

## Abstract

**Background:**

The WALANT (wide-awake local anesthesia with no tourniquet) technique was based on local infiltration of lidocaine and epinephrine. This technique has rapidly gained popularity in recent years and can perform most hand operations. This study aimed to investigate the time spent on anesthesia and operation and perform an economic analysis among general anesthesia, wrist block with a tourniquet, and the WALANT technique for the internal fixation of metacarpal fractures.

**Methods:**

We retrospectively reviewed all the single metacarpal fractures managed with the same procedure, open reduction, and internal fixation with the plate between January 2015 and December 2019. They were divided into three groups according to the method of anesthesia: (1) general anesthesia (GA group), (2) wrist block with a tourniquet (WB group), and (3) WALANT technique (WALANT group). We collected and analyzed patient demographic data, perioperative or postoperative complications, number of hospital days, and postoperative functional recovery assessment.

**Results:**

A total of 63 patients met the inclusion criteria, including 24 in the GA group, 28 in the wrist block group using a tourniquet, and 11 in the WALANT group. There were no complications during the operation and follow-up in each group. The GA group had an average of 32.8 min of anesthesia time, significantly longer than the other two groups. However, there is no significant difference regarding surgical time among the presenting three groups. The discomfort of vomiting and nausea after surgery occurred in 20 patients in the GA group (38.1%). Nevertheless, there was no postoperative vomiting and nausea present in both the WB and WALANT groups. Most patients achieved full recovery of pre-injury interphalangeal and metacarpophalangeal motion at the final assessment of functional recovery.

**Conclusions:**

The patients undergoing metacarpal fixation surgery under WALANT or WB had significantly less anesthesia time and postoperative vomiting and nausea. Moreover, there was no difference in surgical time and intraoperative complications. The time-related reduction improved the utilization of the operation room for additional cases. The reduction of the preoperative examination, anesthesia fee, postoperative recovery room observation, and hospitalization can effectively reduce medical costs. Furthermore, the WALANT group is more acceptable because of no tourniquet, which commonly causes discomfort.

## Background

Traditional hand surgery is performed with a tourniquet to create a bloodless field. The patient must tolerate the discomfort caused by the tourniquet throughout the procedure. Therefore, the surgery is often performed under heavy sedation, such as general anesthesia or regional block, so the patient does not feel surgical pain and discomfort from the tourniquet. Donald et al. [1, 2] introduced the WALANT (wide-awake local anesthesia with no tourniquet) technique in 2005 based on local infiltration of lidocaine and epinephrine. This combination allows the surgeon to use local anesthetics agents to achieve pain-free surgery. The vasoconstriction effect of epinephrine provides adequate hemostasis in the operative field, eliminating the need for a tourniquet.

Epinephrine mainly provides vasoconstriction, so it is not routinely used with a local anesthetic agent during finger surgeries to avoid necrosis. However, the literature has proven that epinephrine is safe in hand surgery, even on the end of the finger [3–6]. In addition to safety, several studies have approved the cost-effectiveness of WALANT [7–9]. Most hand operations can be performed with WALANT today. This technique has rapidly gained popularity in recent years, such as flexor tendon repairs, carpal tunnel release, trigger finger release, ganglion excision, Dupuytren contracture, and wrist arthroscopy [10, 11].

Metacarpal fractures are one of the most common hand injuries encountered in clinical practice. A single metacarpal fracture can often be treated conservatively. However, some cases require surgical treatment of the injured digit, such as rotational malalignment, significantly displaced or angulated fractures, and multiple metacarpal shaft fractures [12]. When performing an internal fixation, one of the following anesthesia types is regularly used to make the procedure operate efficiently, including general anesthesia, regional nerve block, Bier block, and WALANT. Unlike in general anesthesia, the patients who undergo the WALANT technique do not need sedation through monitored anesthesia care, eliminating the need for monitoring by an anesthesia staff and additional examination for pre-anesthesia preparation. Patients do not need to be awake compared to deep sedation and do not experience pain and swelling because of the tourniquet. All the benefits of WALANT reduce medical resources and further shorten the patient’s hospital days, which have subsequently less cost [13–15].

To our knowledge, there are no articles that compare the WALANT technique used for metacarpal fixation with the other anesthesia types. This study aimed to investigate the time spent on anesthesia and operation and perform an economic analysis among general anesthesia, wrist block with a tourniquet, and the WALANT technique for the internal fixation of metacarpal fractures. We hypothesized that WALANT surgery would significantly decrease hospital time and total costs.

## Methods

We retrospectively reviewed all the metacarpal fracture cases with internal fixation with a plate, from a prospectively recorded database, between January 2015 and December 2019. They were divided into three groups according to the method of anesthesia: (1) general anesthesia (GA group), (2) wrist block with a tourniquet (WB group), and (3) WALANT technique (WALANT group). At a level I trauma center (Kaohsiung Veterans General Hospital, Taiwan), we included all the metacarpal fractures with a single finger involvement managed with the same procedure, open reduction, and internal fixation with the plate. Three orthopedic surgeons who specialized in hand surgery performed the procedures. The Institutional Review Board of Kaohsiung Veterans General Hospital approved this study. Patients for all surgical procedures provided written consent for the possible use of anonymized photographs.

The exclusion criteria included the following: (1) patients who had multiple-digit fractures or other concomitant procedures, such as wrist ligament repair or distal radius fracture; (2) deformity or functional impairment of ipsilateral hand before this injury; (3) open fracture or combined with soft tissue loss which may need a secondary procedure; and (4) multiple trauma involving other organs need further procedure at the same time. All enrolled patients had been followed for more than 12 months. The authors collected patient demographic data based on medical records, dates of trauma and surgery, perioperative or postoperative complications, number of hospital days, and follow-up duration based on reviewing medical records. The postoperative functional recovery assessment is based on the patient’s subjective perception of returning to the pre-injury range of motion, including the DIP, PIP, and MCP joints.

### Anesthesia procedure-all groups undergo draping in the standard sterile procedure and the surgical manner

#### GA group

Patients undergoing GA had a tourniquet on the upper arm. After the tourniquet pressure increased to 230 mmHg, the surgeon made an incision for subsequent reduction and fixation. No drugs were injected into the surgical area until the wound was closed.

#### Wrist block with tourniquet group

In this group, the median nerve, ulnar nerve, and superficial radial nerve were anesthetized at the wrist level for a wrist block, using 2% Xylocaine 4 ml, 4 ml, and 2 ml, respectively. An elastic band was applied as a tourniquet at the wrist region and tightened once the patient had no pain in the surgical area. No drugs were injected into the surgical area until the wound was closed.

#### WALANT group

For patients undergoing WALANT surgery, 1% lidocaine with 1:400,000 epinephrine was mixed. It is essential to select the smallest needle when needling to prevent discomfort. Furthermore, forward the needle and administer the regimen as slowly as possible, and press proximal to the planned incision to create sensory noise. A total of 10–20 ml of this mixture was injected into the planned incision line to achieve the tumescent effect. Moreover, an additional 3 ml of the mixture was injected into the palpable displaced fracture site to obtain a hematoma block. After waiting for 18 min, the surgeon made the incision, which is our standard for this formula, to achieve an excellent hemostatic effect.

### Cost savings analysis

With the standard cost estimation protocol, the National Health Insurance (NHI) system in Taiwan was used to determine the efficiency and cost among the three groups. When performing each procedure, the system has a specific fee for the whole treatment, including examination, surgery, surgical equipment, staff cost, and admission. The difference in cost for performing procedures among GA, wrist block, and WALANT indicates cost savings. The preoperative evaluation cost, including electrocardiography, X-ray, routine laboratory examination, anesthesiologist visit, and assessment, were recorded. We also evaluated the surgery’s time cost, such as the time required to prepare for surgery (anesthesia time) and the time required to finish the surgery (surgical time).

### Statistics

The SPSS 22.0 statistical software package (SPSS Inc., Illinois, USA) analyzed the data. The Fisher’s test was applied for categorical data (sex, vomiting and nausea discomfort, and recovery to full range of motion). The Kruskal–Wallis test was performed for non-parametric continuous data such as age, time, and pain scale. A P value < 0.05 was considered statistically significant. Furthermore, we analyzed the pairwise comparison if the Kruskal–Wallis test had significant findings.

## Results

A total of 63 patients met the inclusion criteria with a single metacarpal fracture (Table 1). All patients aged 17–87 years underwent an operation of open reduction with plate fixation. The patients were divided into three groups: 24 in the GA group, 28 in the wrist block group using a tourniquet, and 11 in the WALANT group. The average ages were 36.7 ± 17.1, 41.9 ± 19.0, and 41.5 ± 23.7 years for the GA, WB, and WALANT groups. There were 9 females in the GA group (37.5%), 7 in the WB group (25%), and 3 in the WALANT group (27.3%). There were no complications during the operation and follow-up in each group. None of these patients had malrotation-induced scissoring deformity or nonunion or loss of reduction, which need secondary surgery. None of the patients in the WB and WALANT groups required a further intraoperative lidocaine bolus.

**Table 1:** Demographic data and clinical result

	GA group (n = 24)	WB group (n = 28)	WALANT group (n = 11)	*P* value
Age	36.67(17–62)	41.86(18–76)	41.55(19–87)	0.445*
Sex, M:F	15:9	21:7	2:9	0.606^+^
ER to OR time, days	2.29(0–8)	1.39(0–8)	2.0(0–9)	0.302*
Anesthesia time, min	32.83(15–60)	19.46(9–45)	21.36(18–25)	< 0.001*
GA v.s. WB				< 0.001
GA v.s. WALANT				0.003
Surgical time, min	44.67(15–119)	43.68(20–110)	50.72(15–98)	0.315*
Discomfort of vomiting and nausea	20 (38.1%)	0	0	0.017^+^
Postop day 1 VAS	2.92(1–5)	2.71(1–5)	2.18(1–3)	0.056*
Full ROM recovery	21(87.5%)	27(96.42%)	11(100%)	0.214^+^

*GA* general anesthesia, *WB* wrist block, *WALANT* wide-awake local anesthesia no tourniquet, *VAS* visual analog scale, *ROM* range of motion

*Fisher’s exact test

^+^Kruskal–Wallis test

**Fig. 1:**
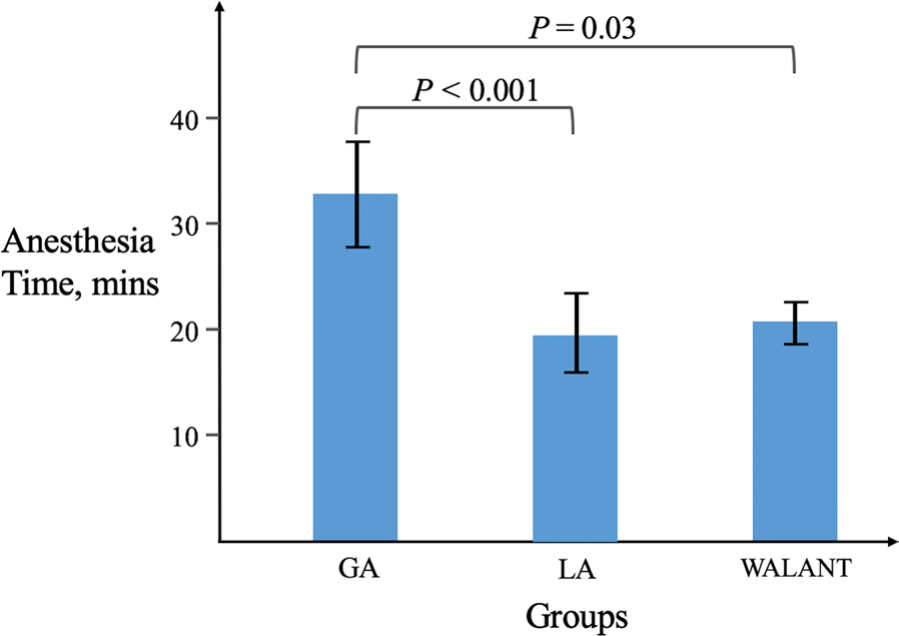
Pairwise comparison among the three groups following the Kruskal–Wallis test of anesthesia time

Regarding the perioperative and postoperative variables (Table 1), specifically the emergency room to operation room (OR) time, as defined by the time (day) between diagnosis in the emergency room and the time to operation, patients in the GA, WB, and WALANT groups averaged 2.3, 1.4, and 2.0 days (*P* > 0.05), respectively. Regarding anesthesia time, patients in the GA, WB, and WALANT groups had an average of 32.8, 19.5, and 21.4 min, respectively. The GA group had a statistically significant difference longer than the other two groups (Fig. 1). Regarding surgical time, defined as the documented incision start and wound closure, the GA, WB, and WALANT groups had an average of 44.7, 43.7, and 50.7 min, respectively (P > 0.05). The discomfort of vomiting and nausea after surgery in these groups occurred in 20 patients in the GA group (38.1%). However, there was no postoperative vomiting and nausea present in both the WB and WALANT groups (P < 0.05). The patients’ subjective perceptions of pain on the 1st day after surgery averaged 2.9, 2.7, and 2.18 in the GA, WB, and WALANT groups. Although there is less pain in the WALANT group than the other two, it is statistically significant. For the final assessment of functional recovery after more than 1 year. Most patients achieved full recovery of pre-injury interphalangeal and metacarpophalangeal motion (87.5% of the GA group, 96.4% of the WB group, and 100% of the WALANT group).

According to the Taiwan NHI system, each management and examination has a corresponding price. We analyzed what each patient performed under GA will have excess spend on clinical resources compared to both WB and WALANT patients, including ward fee, nursing fee, pre-operation anesthesia consultation, lab work, chest X-ray, electrocardiography, medication and supplement of GA, and a tourniquet (Table 2).

**Table 2:** Additional cost via general anesthesia

Types of cost		Cost, point
Preoperative evaluation		1370
	Anesthetic evaluation	
	Lab work	
	Chest x-ray	
	Electrocardiography	
Anesthesia		5373
	Technical fee, less than 2 h	
	Equipment and medication	
Hospitalization		1300
	Ward fee*	
	Nursing fee*	
	Fee for postoperative care unit	
Total		8043^+^

*Daily fee

^+^After conversion, it is approximately equivalent to 287 USD

## Discussion

Metacarpal bone fracture is a common hand injury often treated with open reduction and internal fixation with a plate or percutaneous pinning. Anesthetic practices, including sedation with GA, have been utilized during surgery for sedation and pain control for surgical pain and tourniquet discomfort. Alternatively, nerve blocks could be used, such as a wrist block or an ultrasound-guided nerve block at a high level. Between 1920 and 1940, the use of procaine led to a necrotic finger at a very acidic status due to more extended storage [6, 16]. However, this was attributed to epinephrine because of the vasoconstriction effect. The concern related to the safety of epinephrine arose until recently. Since Donald et al. proposed wide-awake surgery and epinephrine safety in the hand [1, 3, 4], the WALANT surgery was widely used in all kinds of hand surgery. The concept is that xylocaine can achieve pain relief and use a safe epinephrine dose to constrict bloody oozing in the surgical area, even without a tourniquet, to control bleeding for a smooth operation. Literature has shown that WALANT offered patients and surgeons an effective and alternative way for surgical treatment of the metacarpal fractures.

Most metacarpal internal fixation has been performed under general or wrist block with a tourniquet in our hospital. In our analysis of different anesthesia approaches via GA, WB, and WALANT in treating metacarpal fracture, we found no significant difference in the surgical time spent from incision to wound closure. However, our results did demonstrate significantly less anesthesia time in both the WB and WALANT groups. Once the patient arrives in the operating room, we immediately prepared the anesthesia process, including the injection for pain relief and later hemostasis, followed by sterilization and instrumental preparation. There is no need to wait for the arrival of an anesthesiologist and associated staff and the time for induction and intubation. The surgical field certainly had almost no bleeding under the tourniquet in the GA and WB groups when performing the procedure. WALANT surgery relies on epinephrine to stop bleeding so that the surgical field of vision is clear and can be completed smoothly alongside fracture restoration and fixation. Nevertheless, the procedure still requires ligation for larger vessels. There would only be a little bit of bleeding after enough waiting for the hemostatic effect from the specific regimen, mainly from hematoma accumulation at the fracture site. Usually, a 3 × 3 in gauze was enough to absorb all bleeding, so we did not record and compare the amount of blood loss. In that case, the operation time was not longer than the other two groups.

In this study, a significantly higher incidence of postoperative vomiting and nausea events in the GA group was noted. The patients in both WALANT and WB groups did not receive anesthetic agents for deep sedation, and thus, they do not have to recover from their side effects such as nausea, vomiting, and dizziness. Patients were able to go home right after surgery without a need for monitoring postoperative recovery [5]. According to the pain scale on postoperative day 1, we found that the WALANT group was not significantly different from the other two groups. However, there was still a trend toward a lower pain scale because not using a tourniquet allowed the patient’s postoperative pain to be confined to the surgical area and not extend to the wrist or upper arm. Furthermore, there was no reperfusion caused by deflating the tourniquet leading to swelling and ecchymosis. This is beneficial to the patient’s pain perception. Both the general discomfort and surgical pain after surgery indeed cause dissatisfaction in patients. Nevertheless, most of our patients could return to their preoperative range of motion level without significant differences among the three groups. The outcome of fracture surgery usually depends on the type of fracture, articular surface involvement, an associated injury, implant choice, and whether the reduction is successful. All the above factors were controlled in this presenting study, and therefore there were no significant differences.

Since the preoperative testing, anesthesia fee, and hospitalization are all eliminated, higher cost savings are associated with WALANT technique [7, 9]. Instead of GA, WALANT surgery for internal metacarpal fixation demonstrated savings of approximately 287 USD per case (Table 2). However, this number represents the apparent cost, such as ward and staff fees, equipment, and examination. It underestimates the actual cost savings associated with WALANT surgery, including drugs for pain control or anesthesia, anesthesia set-up costs, and equipment maintenance costs typically incurred by facilities that provide anesthesia services. In addition, with less anesthesia time and no time needed to recover from sedation, patients under WALANT spent less time in the operating room, which made the arrangement and performance of all surgical procedures more efficient.

This presenting study has several limitations. First, this is a retrospective design study based on the experience of a single institution. Second, we only considered single metacarpal surgery to decrease bias, excluding revision surgery or surgery combined with other procedures. Thus the number of patients in each group was small. Especially for the WALANT group, the groupings in this study were based on patients’ preferences. Some were nervous about having to be awake for the fracture surgery after explaining the WALANT procedure. Third, this study was designed to investigate perioperative times spent and economic analysis. There was no specific assessment questionnaire that compared the functional differences among groups, as most patients can return to full function regardless of the anesthetic approach. Last, the inability of the Taiwan NHI to account for all direct and indirect costs for performing a procedure results in the underestimation of the cost savings of the WALANT technique. In addition, the Taiwan NHI is also part of the social welfare system. The government introduces resources to support it, so the value of each medical service cannot be directly correlated to different countries. It can only be compared to other groups in the present study to indicate cost-effectiveness.

## Conclusions

In contrast to GA, patients undergoing metacarpal fixation surgery under WALANT or WB had significantly less anesthesia time and postoperative vomiting and nausea. Moreover, there was no difference in surgical time from incision to closure and intraoperative complications. The time-related cost reduction improved the utilization of the OR for additional cases. The reduction of the preoperative examination, anesthesia fee, postoperative recovery room observation, and hospitalization can effectively reduce medical costs. Furthermore, the WALANT group is more acceptable to patients than the WB group because they do not need to use a tourniquet, which commonly causes discomfort.

## Data Availability

The datasets used and analyzed during this study are available from the corresponding author on reasonable request.

## Abbreviations

WALANT: Wide-awake local anesthesia with no tourniquet
GA: General anesthesia
WB: Wrist block
NHI: National health insurance
MCP: Metacarpophalangeal
PIP: Proximal interphalangeal
DIP: Distal interphalangeal
OR: Operation room
